# CBCT Evaluation of the Upper Airway Morphological Changes in Growing Patients of Class II Division 1 Malocclusion with Mandibular Retrusion Using Twin Block Appliance: A Comparative Research

**DOI:** 10.1371/journal.pone.0094378

**Published:** 2014-04-04

**Authors:** Liang Li, Hong Liu, Huijuan Cheng, Yanzhao Han, Chunling Wang, Yu Chen, Jinlin Song, Dongxu Liu

**Affiliations:** 1 Department of Orthodontics, Shandong Provincial Key Laboratory of Oral Biomedicine, School of Dentistry, Shandong University, Jinan, China; 2 Department of Orthodontics, School of Dentistry, Shandong University, Jinan, China; 3 Department of Orthodontics, Shandong Provincial Key Laboratory of Oral Biomedicine, School of Dentistry, Shandong University, Jinan, China; 4 Department of Orthodontics, Chongqing Key Laboratory for Oral Diseases and Biomedical Sciences, the Affiliated Hospital of Stomatology, Chongqing Medical University, Chongqing, China; The Ohio State University, United States of America

## Abstract

**Objective:**

The purpose of this study was to evaluate the morphological changes of upper airway after Twin Block (TB) treatment in growing patients with Class II division 1 malocclusion and mandibular retrusion compared with untreated Class II patients by cone beam computed tomography (CBCT).

**Materials and Methods:**

Thirty growing patients who have completed TB treatment were recruited into TB group. The control group (n = 30) was selected from the patients with the same diagnosis and without TB treatment. CBCT scans of the pre-treatment (T1) and post-treatment (T2) data of TB group and control data were collected. After three-dimensional (3D) reconstruction and registration of T1 and T2 data, the morphological changes of upper airway during TB treatment were measured. The statistical differences between T1 and T2 data of TB group as well as T2 and control data were accessed by t-test.

**Results:**

During the TB treatment, the mandible moved advanced by 3.52±2.14 mm in the horizontal direction and 3.77±2.10 mm in the vertical direction. The hyoid bone was in a more forward and inferior place. The upper airway showed a significant enlargement in nasopharynx, oropharynx and hypopharynx. In addition, the nasopharynx turned more circular, and the oropharynx became more elliptic in transverse shape. However, the transverse shape of the hypopharynx showed no significant difference. After comparison between T2 and control data, only the horizontal movement of the hyoid bone, the volumetric expansion of the oropharynx and hypopharynx, and changes of the oropharyngeal transverse shape showed significant difference.

**Conclusion:**

Compared to the untreated Class II patients, the upper airway of growing patients with Class II division 1 malocclusion and mandibular retrusion showed a significant enlargement in the oropharynx and hypopharynx as well as a more elliptic transverse shape in the oropharynx, and the hyoid bone moved to an anterior position after TB treatment.

## Introduction

Narrowing of the upper airway has been increasingly recognized as a physiological characteristic in growing patients of Class II division 1 malocclusion with mandibular retrusion [1]–[3]. The retarded mandible induces a retro-displacement of the tongue and hyoid bone that may lead to a concomitant reduction in the upper airway volume [2], [4]. Many studies have demonstrated that the airway constriction is the most dominating contributor to obstructive sleep apnea (OSA), which affected at least 2% of children and was confirmed to have a long-term adverse affects [5]–[7]. Therefore, detailed assessment of the upper airway is essential for routine orthodontic planning and treatment outcome evaluation for this type of malocclusion.

The Twin Block (TB) appliance is a well-accepted approach in correcting Class II division 1 malocclusion with mandibular retrusion in recent years. Previous studies indicated that TB is effective in mandibular forward repositioning and thereby achieves a more harmonious facial profile [8], [9]. The upper airway changes in adult patients with OSA were measured after mandibular repositioning treatment, and showed a significant enlargement in pharyngeal volume [10], [11]. However, Lin et al. [12] evaluated the pharyngeal airway after mandibular advancement in growing patients with retrognathia, and suggested that the anteroposterior dimension of pharyngeal airway did not change significantly. The majority of the studies examined the changes of the upper airway through lateral cephalometric radiography. This method limited the accuracy of airway measurement since the two-dimensional (2D) images only allowed an anteroposterior dimension measurement in sagittal plane, and failed to provide a full-scaled view of the upper airway [13]. Therefore, retrospective 3D evaluation of the upper airway in growing patients during TB treatment needs to be established.

Cone beam computed tomography (CBCT) is an acceptable technique for 3D volumetric depiction and morphological evaluation of the upper airway using a lower-radiation method with a greater spatial resolution [14]. It allows 3D registration of pre- and post-treatment data through identification of specific structures in the cranial base. Therefore, the changes of the upper airway before and after mandibular advancement can be precisely evaluated [15], [16].

As the patients involved in our research were underage, their normal development may affect on the upper airway morphology during the treatment period. Thus, an untreated control group is needed. In the present study, the control group was selected from patients with the same diagnosis who just began orthodontic treatment, and matches well with the post-treatment patients of TB group through age, sex and development condition. Using this strategy, effects of TB on the upper airway morphology can be evaluated.

The purpose of this study is to use retrospective 3D registration, to evaluate the changes of the upper airway in growing patients with Class II division 1 malocclusion and mandibular retrusion during TB treatment.

## Materials and Methods

### Ethics Statement

Human subjects participated in the research after parents or guardians signed the written informed consent in accordance with the study protocol approved by the Research Ethic Committee of Shandong University Dental School (protocol number 2013056).

### Subjects

The same selection criteria were used for both TB and control groups: 1) the overjet was at least 7 mm with an Angle Class II molar relationship; 2) SNA angle was within the normal range between 78.4° and 85°; 3) SNB angle was less than 75°, and the patients had a clinically retrognathic mandible which is determined by the soft tissue of the chin being posterior to Bass' analysis vertical reference line [17]; 4) ANB angle was greater than 4°; 5) the patients had no other potential airway abnormalities.

Thirty growing patients who have completed TB treatment between 2011 and 2013 were included in this study as TB group (13 boys and 17 girls, mean age 11.57±0.94 years). The control group (13 boys and 17 girls, mean age 11.72±0.86 years) was selected to match with TB group based on age, sex and development condition.

**Figure 1:**
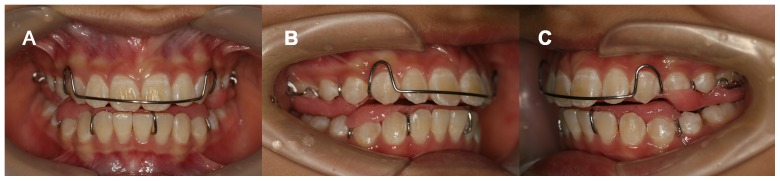
Patients of TB group were instructed to wear TB appliance. A) The front view. B) The right view. C) The left view.

The treatment duration of TB group was 13.67±1.51 months. During TB therapy, patients of TB group were instructed to wear the TB appliance for 24 hours a day except during sports activities and tooth brushing (Figure 1A–C). The patients were examined at 1-month intervals until the functional appliance therapy completed.

### CBCT Data Acquisition

The CBCT scans of the pre-treatment and post-treatment of TB group were collected as T1 data and T2 data, respectively. The CBCT scans of control group were collected before orthodontic therapy as control data.

During CBCT scanning, patients were instructed to maintain an upright sitting posture and natural head position. The rest position of the tongue (in contact with anterior palate without touching the anterior teeth) and maximum intercuspation were also required. All of the scans were performed by the same researcher.

Images were acquired using the CBCT scanner (KaVo Dental GmbH, Bismarckring, Germany) at a 0.30-voxel resolution with the scanning parameter of 120 kV, 5 mA. The scan time was 8.9 seconds, and the slice thickness was 0.4 mm. The CBCT datasets were exported in the DICOM (Digital Imaging and Communications in Medicine) format.

### 3D Virtual Model Reconstruction

The CBCT data were transferred into MIMICS 16.0(Materialism's Interactive Medical Image Control System) software package. Then the patient-specific 3D models of the upper airway, mandible, hyoid bone and vertebra were reconstructed through segmentation and separate from neighboring tissues by the threshold based on Hounsfield Units (HU).

**Figure 2:**
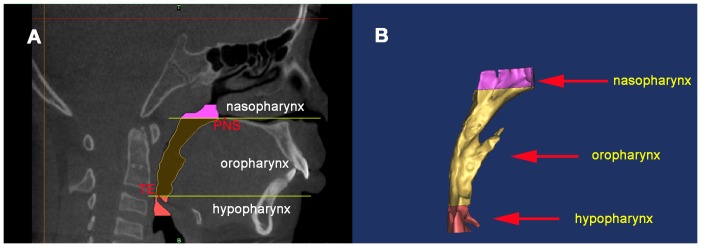
3D model of the upper airway was reconstructed. **A**) The upper airway was subdivided into three parts by two planes perpendicular to the sagittal plane and each region was highlighted in different colors. The landmarks used for defining the planes were: PNS, posterior nasal spine; SE, the superior border of the epiglottis. **B**) Each region of the upper airway was reconstructed respectively. The nasopharynx is the region from the top of the upper airway to PNS. Oropharynx region is between PNS and SE, and the hypopharynx is from SE to the level of the third cervical vertebra (C3).

After identification of the PNS point (posterior nasal spine), the superior border of the epiglottis and the C_3_ point (the third cervical vertebra) in the midsagittal plane, the upper airway was divided into three parts: the nasopharynx, oropharynx, and hypopharynx by the corresponding cross-sectional slices. The nasopharynx is the region from the top of the upper airway to posterior nasal spine, the oropharynx is located between posterior nasal spine and the superior border of the epiglottis, and the hypopharynx is defined as the region from the superior border of the epiglottis to the level of C_3_ point. Each region was also reconstructed in MIMICS (Figure 2A–B). All the 3D models were exported as stereolithography (STL).

### Registration of Pre- and Post-treatment Models

**Figure 3:**
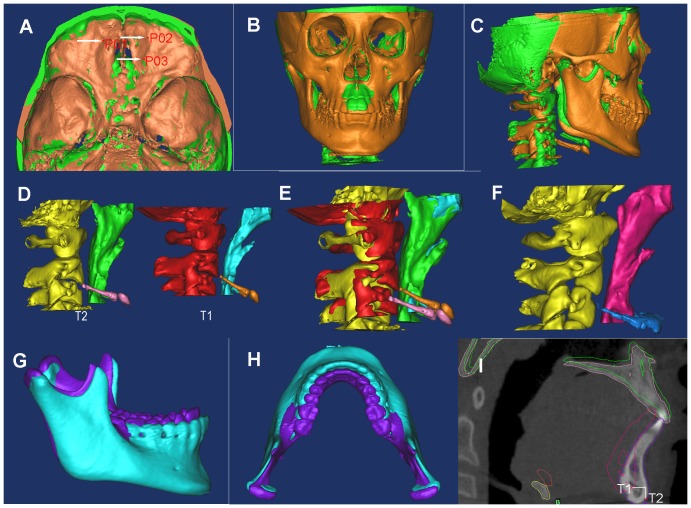
The pre- (T1) and post-treatment (T2) data of TB patients was registered. **A**) Point registration with the most protruding points on the anterior cranial base. **B**) STL registration of T1 and T2 models in front. **C**) STL registration of T1 and T2 models in lateral. **D**) The upper airway models of T1 and T2 data. **E**) STL registration of the upper airway of T1 and T2 data. **F**) The upper airway model of the control data. **G**) STL registration of the mandible of T1 and T2 data in the sagittal view. **H**) STL registration of the mandible of T1 and T2 data in the axial view. **I**) Displacement of the mandible during TB treatment was measured.

In MIMICS, the T2 STLs of TB group was registered with the T1 3D models by point-registration based on the anterior cranial base structures which have completed growth by age of 7 [18], [19]. The registration was accomplished by placing landmark points [20] on the STL images and 3D models which represent the same anatomic sites. MIMICS calculated the transformation matrix for the best fit between the start-end points and then applied it to the selected one. After point registration, STL registration was performed to place STL images on CBCT masks in order to improve the accuracy. Corresponding landmarks were identified repeatedly to ensure the precision (minimal point distance filter was 0.10 mm, which was considered as satisfactory, as in Figure 3A–C).

### 3D measurement

**Figure 4:**
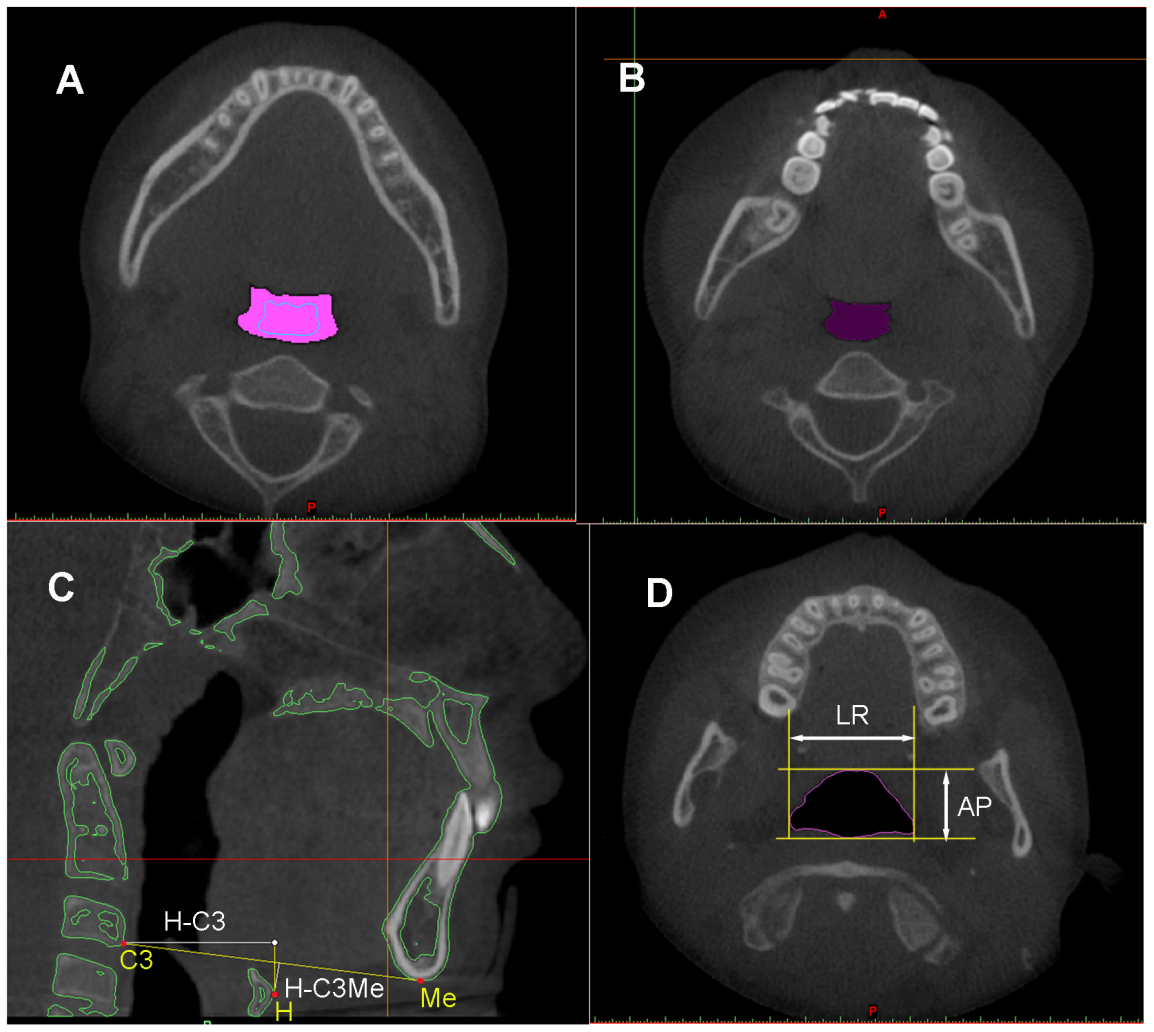
The pharyngeal morphology and the hyoid bone were measured. **A**) Changes of the upper airway between T1 and T2 data in the axial view. **B**) The upper airway of the control data in the axial view. **C**) Horizontal displacement of the hyoid (H) relative to C3, and the perpendicular distance of H to the line connecting C3 and menton (Me) were measured. **D**) The largest anteroposterior (AP) and lateral (LR) dimensions for each cross-sectional slice were measured.

After the reconstruction of each region of the upper airway, MIMICS software automatically calculated the volume (V) and height (H). The mean cross-sectional area (mCSA) was computed as the ratio of V/H. The largest anteroposterior (AP) and lateral (LR) dimensions for each cross-sectional slice were measured in the axial section (Figure 4D). Then, the LR/AP ratio was calculated to determine the shape of the upper airway. The cross section turns more elliptic when the ratio becomes larger, while it turns more circular if the ratio becomes smaller. After the completion of registration, the morphology changes of each region from nasopharynx to hypopharynx were evaluated in 3D models and sagittal slices (Figures 3D–F, 4A–B).

In the TB group, the horizontal displacement of hyoid bone was measured from hyoid (H) to C_3_ point (HC_3_) in the midsagittal plane, and its vertical position was evaluated through the perpendicular distance between H and C_3_-menton line (H-C_3_Me) (Figure 4C) [21], [22]. The displacement of the mandible (Gn) was measured in horizontal (X-Gn) and vertical (Y-Gn) directions, respectively (Figure 3G-I). All the variables in this group were measured in T1 data and T2 data, respectively. After the measurements, the change amount between T1 and T2 was calculated. The landmarks identified on each CBCT model were listed in Table 1. Variables measured on each 3D model are shown in Table 2. All the patients are measured by the same researcher. The measurements were repeated three times, and then the average value was used in our study.

**Table 1:** Definition of landmarks used in this study.

Symbol	Definition
Gn	The midpoint between the most anterior and inferior points on the outline of the mandibular symphysis
Me	The most inferior point on the outline of the mandibular symphysis
H	The most anterior and inferior point of the hyoid bone
C_3_	The most anterior and inferior point of the third cervical vertebra
C_3_-Me line	The line connected between point C_3_ and point Me
CSA	The cross-sectional area of the upper airway on each CBCT sagittal plane

**Table 2:** Definitions of airway measurements variables.

Symbol	Description	Definition
V	volume	Volume of each region of the upper airway (nasopharynx, oropharynx and hypopharynx)
mCSA	Mean cross-sectional area	Mean cross-sectional area of each region of the upper airway
HC3	Hyoid bone to the third cervical vertebra	Distance from H to C_3_ in the sagittal plane
H-C3Me	Hyoid bone to the C_3_-Me line	Perpendicular distance between H and C_3_-Me line
X-Gn	Mandible advancement in the horizontal direction	The mean amount of mandibular advancement at Gn in the horizontal direction
Y-Gn	Mandible advancement in the vertical direction	The mean amount of mandibular advancement at Gn in the vertical direction
LR	Lateral dimension	The lateral dimension of the pharyngeal on each CBCT axial plane
AP	Anteroposterior dimension	The anteroposterior dimension of the pharyngeal on each CBCT axial plane
LR/AP	Ratio of LR to AP dimensions	Ratio of LR/AP dimensions

### Statistical Analysis and Measurement Reliability

The statistical analysis was performed using the SPSS 17.0. The mean value and standard deviation (SD) was calculated for every variable measured in the 3D models. The changes of hyoid bone position and LR/AP ratio in each region of the upper airway during TB treatment were analyzed by paired-t test. The independent t-test was used for comparisons between T2 and control data.

All measurements were repeated for every patient, with a 1-month interval, to assess intra-rater reliability. The statistical differences between the original and repeated measurements were determined by paired t-test at the significance level of 0.05. The method error (ME) was calculated as: ME = 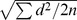 (d is deviation between the two measurements; n for the number of paired double measurements) [23].

## Results

The age distribution between the post-treatment patients of TB group and patients of control group showed no significant difference (*P* = 0.812). No statistical differences were found between the original and repeated measurements (*P* = 0.903). The method error varied from 0.03 to 0.14 mm for 3D liear measurement and from 5.76 to 7.85 mm^2^ for measurement of the upper airway cross-sectional area.

The measurement variables were listed in Table 3. In the TB group, the mandible advanced by 3.52±2.14 mm (mean±SD) in the horizontal direction and 3.77±2.10 mm in the vertical direction, respectively. With the mandibular advancement, the hyoid bone showed a more forward and downward displacement, and the upper airway expanded after treatment. The greatest changes of volume and mean cross-sectional area occurred in the oropharynx. In addition, the post-oropharynx turned more elliptic in the transverse shape compared with the pre-treatment while the post-nasopharynx became more circular. The hypopharyngeal shape, however, showed no significantly change during treatment.

**Table 3:** The measured variables in TB group and control group.

	TB (n = 30)				Control (n = 30)		p value	
	T1		T2					
Variables	mean	SD	mean	SD	mean	SD	T1&T2	T2&control
H-C3	27.22	2.54	30.18	3.24	28.26	2.52	.022	.017
H-C3Me	2.05	1.95	4.32	2.18	4.86	2.04	.045	.105
Na-V	3086.42	381.67	3662.40	463.72	3638.72	493.05		.865
Na-mCSA	288.94	47.02	308.10	42.11	304.46	46.32		.415
Na-LR/AP	2.15	0.17	1.77	0.33	1.72	0.76	.047	.691
Or-V	5348.98	521.52	7075.06	683.16	6368.75	632.92		.028
Or-mCSA	161.80	30.58	195.21	37.95	181.61	41.69		.010
Or-LR/AP	1.87	0.31	2.27	0.49	1.96	0.68	.013	.032
Hy-V	1764.77	173.99	2264.20	135.75	2074.36	108.84		.018
Hy-mCSA	192.52	29.29	219.04	27.25	205.03	35.27		.043
Hy-LR/AP	2.55	0.71	2.42	0.41	2.45	0.58	.125	.235

Na, nasopharyngeal; Or, oropharyngeal; Hy, hypopharynxgeal.

By comparing the T2 data of TB group and the control data, we discovered that the hyoid bone in the post-TB group was more anterior but its vertical position did not show a significant difference. A significant enlargement in the oropharynx and hypopharynx after TB treatment was observed. In addition, the oropharynx showed more oval-shaped after TB treatment.

## Discussion

In this study, we investigated the effects of TB on the upper airway morphology in growing patients with Class II division 1 malocclusion and mandibular retrusion in compared with the controls through 3D reconstructive assessment. After TB treatment, the oropharynx and hypopharynx expanded significantly compared to the control group, and the oropharynx had the maximum change in volume and cross-sectional area. Besides, oropharyngeal shape turned more elliptical than control group, indicating that the main effect of the TB appliance was on the oropharynx. Therefore, TB appliance may improve the treatment for airway obstruction, which mostly occurred in the oropharynx [3], [24].

Previous literatures reported obvious dimensional changes in the oropharynx following mandibular advancement treatment [1], [11], [13], suggesting that the patency of the oropharynx was attributed to a forward repositioning of the tongue and soft palate with the mandibular advancement. In the present study, the hyoid bone showed a consistent forward movement with the mandible relative to the C_3_ level, indicating that the hyoid bone might be a contributing factor to the oropharynx enlargement. Muscles connected with the tongue or mandible to the hyoid might pull the hyoid anteriorly when the mandible and tongue were more advanced [25].

Eggensperger et al. [26] discovered a significant correlation between the horizontal change in position of the hyoid bone and mandibular movement in a long-term follow-up study of mandibular advancement treatment, and confirmed that the horizontal movement of the hyoid bone might be an indicator of the mandibular translocation. Several studies [6], [27] have evaluated the sagittal position of hyoid bone in children with OSA, and reported that the distance between hyoid bone and C_3_ is larger in these patients as a result of compensatory reaction to improve respiratory pattern, demonstrating that the hyoid bone is indicative of the pharyngeal ventilatory function. A relationship between the position of hyoid bone and the hypopharyngeal dimension has been reported [28], [29], which is in accordance with our study that the hypopharynx of the post-treatment patients in TB group is increased following the forward movement of the hyoid bone.

The vertical position of the hyoid bone in the post-TB group data showed no significant difference from the control data, indicating that growth development plays a vital role in vertical control of the hyoid bone. Sheng et al. [30] analyzed the hyoid bone position through a longitudinal study from the mixed-dentition period to the young-adult stage, and found that the hyoid bone moved downward as the age grows. In the present study, as a result of the restriction drawn by the posterior belly of the digastric muscle and the infra-hyoid muscles [14], [31], the post-hyoid showed a more posterior trend relative to the pre-hyoid position. However, the C_3_ point was also unstable and turned more backward during TB treatment. Therefore, HC_3_ is a proper variable to describe the horizontal displacement of hyoid bone for growing patients.

The post-oropharynx became more elliptical in cross section than before, which agreed with the previous findings [32], [33], indicating that the mandibular advancement caused a more significant expansion in the lateral oropharyngeal wall than the anterior wall. It was hypothesized that there was a reflex response of the stylopharyngeus muscle to the drag effect upon hyoid bone owing to the mandible forward reposition [14]. Fogel et al. [34] found that a more AP-oriented than laterally oriented airway was positively correlated with severity of sleep apnea. In addition, the reduction of minimum palatal airway width was reported to positively correlate to OSA [35]. Thus, we conclude that a wider oropharynx increases the patency of the upper airway and prevents the OSA compared with a more AP-oriented airway.

The nasopharynx showed a more circular shape in cross section of the post-TB group. This might result from normal development of the patients as no statistical difference was observed comparing to the control group. Other studies also reported that the height and anteroposterior diameter of the nasopharynx increased during development, but the width reminds unchanged [36], [37].

## Conclusion

Comparing to the untreated Class II patients, the oropharynx and hypopharynx of growing patients with Class II division 1 malocclusion and mandibular retrusion showed a significant enlargement and the hyoid bone moved to a more anterior place. In addition, the oropharynx was found to be more elliptic in transverse shape after TB treatment.
